# Antibody-mediated delivery of viral epitopes to redirect EBV-specific CD8^+^ T-cell immunity towards cancer cells

**DOI:** 10.1038/s41417-023-00681-4

**Published:** 2023-11-09

**Authors:** Willemijn van der Wulp, Dennis F. G. Remst, Michel G. D. Kester, Renate S. Hagedoorn, Paul W. H. I. Parren, Sander I. van Kasteren, Janine Schuurman, Rob C. Hoeben, Maaike E. Ressing, Boris Bleijlevens, Mirjam H. M. Heemskerk

**Affiliations:** 1 Department of Cell and Chemical Biology, Leiden University Medical Center, Leiden, the Netherlands; 2 Department of Hematology, Leiden University Medical Center, Leiden, the Netherlands; 3 Department of Immunology, Leiden University Medical Center, Leiden, the Netherlands; 4 Division of Bio-organic Synthesis, Leiden Institute of Chemistry, Leiden University, Leiden, the Netherlands; 5 Genmab, Utrecht, the Netherlands

**Keywords:** Immunization, Cellular immunity, Tumour immunology

## Abstract

Antibody-mediated delivery of immunogenic epitopes to redirect virus-specific CD8^+^ T-cells towards cancer cells is an emerging and promising new therapeutic strategy. These so-called antibody-epitope conjugates (AECs) rely on the proteolytic release of the epitopes close to the tumor surface for presentation by HLA class I molecules to eventually redirect and activate virus-specific CD8^+^ T-cells towards tumor cells. We fused the immunogenic EBV-BRLF1 epitope preceded by a protease cleavage site to the C-terminus of the heavy and/or light chains of cetuximab and trastuzumab. We evaluated these AECs and found that, even though all AECs were able to redirect the EBV-specific T-cells, AECs with an epitope fused to the C-terminus of the heavy chain resulted in higher levels of T-cell activation compared to AECs with the same epitope fused to the light chain of an antibody. We observed that all AECs were depending on the presence of the antibody target, that the level of T-cell activation correlated with expression levels of the antibody target, and that our AECs could efficiently deliver the BRLF1 epitope to cancer cell lines from different origins (breast, ovarian, lung, and cervical cancer and a multiple myeloma). Moreover, in vivo, the AECs efficiently reduced tumor burden and increased the overall survival, which was prolonged even further in combination with immune checkpoint blockade. We demonstrate the potential of these genetically fused AECs to redirect the potent EBV-specific T-cells towards cancer in vitro and in vivo.

## Introduction

Clinical therapies that aim at antibody-mediated redirection of T-cells towards cancer cells are a successful therapeutic strategy for liquid cancers with a few registered products available for patients (e.g. blinatumomab, mosunetuzumab and Tebentafusp) [1–3] or are in process of approval or clinical evaluation [4]. All these make use of the principle to engage CD3^+^ T-cells in the killing of cancer cells and bypass the need for tumor-specific T-cells, however have not been as potent for solid tumors as for hematological cancers [4, 5]. The lower efficacy towards solid tumors can be attributed to several challenges, such as, the quality of the tumor-infiltrating T-cells (TILs) in the tumor microenvironment (TME) and on-target off-tumor toxicities caused by low expression levels of the tumor-associated antigens (TAAs) expressed on healthy tissues [4, 6–8]. Moreover, targeting of all CD3 expressing T-cells may lead to an important side effect: the unwanted and excessive release of cytokines called the cytokine-release syndrome (CRS) [9]. Therefore, it may be beneficial to redirect a more limited group of CD8^+^ T-cells that are known to be very potent instead of the entire CD3^+^ T-cell population.

Some viruses are widely prevalent in the human population such as the human herpesviruses cytomegalovirus (CMV) and Epstein-Barr virus (EBV) [10, 11]. These viruses have coevolved with humans and can persist as a lifelong, (largely) asymptomatic, latent infection with occasional reactivations [12]. In EBV and CMV infections, T-cell immunity plays a pivotal role in the clearance of the virus and can lead to an unusual large number of potent CD8^+^ T-cells [11, 13]. Virus-specific T-cells are present in the TME but can only act as bystanders as there are no target antigens expressed by the tumor [14, 15]. It was previously shown that intratumoral injection of virus-derived peptides can overcome the immunosuppressive TME and can trigger an effective antiviral T-cell response against the tumor [16]. Therefore, this group of T-cells might be attractive candidates to be redirected towards the cancer cells.

To be able to redirect those virus-specific T-cells, immunogenic EBV or CMV T-cell major histocompatibility class I (MHC-I) epitopes were conjugated to tumor-targeting antibodies. With several antibody conjugation and delivery strategies it was proven that antibodies can efficiently deliver viral epitopes to cancer cells [17–21] and we recently compared three approaches to generate these antibody-epitope conjugates (AECs) for cetuximab (CTX) and trastuzumab (TRS) [22]. It was demonstrated that AECs generated by means of a genetic fusion resulted not only in the most well-defined, but also the AECs with the highest stability. In this study, we explored the possibilities to increase the epitope-to-antibody ratio (EAR) and investigated the in vivo functionality of the genetically fused AECs in a xenograft mouse model.

## Materials and methods

### Antibodies and tetramers

Cetuximab (CTX) and pembrolizumab were obtained from Merck (Germany, Darmstadt). Trastuzumab (TRS) and all genetically modified antibodies were produced at Genmab via transient expression in ExpiHEK293 FreeStyle cells as described before [23] and purified by Protein A affinity chromatography. If required, protein aggregates were removed via Size Exclusion Chromatography to yield protein product with *a* > 95% monomeric content as analyzed on HPLC-SEC. All antibodies were stored in phosphate-buffered saline (PBS). For the AECs used in vivo experiments mutations were introduced (P329G, L234A, and L235A) to disrupt possible interactions with Fc-receptors [24].

The following antibodies were used for flow cytometry: cetuximab, trastuzumab, goat anti-human IgG-A488 (Jackson immunoResearch, UK, Cambridgeshire, #109-546-098) or -PE (Jackson immunoResearch, #109-116-098), goat anti-mouse-FITC (Jackson immunoResearch, 115-096-062), mouse anti-HLA-A2 (produced in-house from clone BB7.2 [25]), mouse anti-human EGFR (Santa Cruz Biotechnology, #sc-120), and mouse anti-human Her2 (R&D, UK Abingdon, #MAB9896). Peptide MHC multimers complexes (tetramers) were generated in-house as described before [26] and labeled with PE-conjugated streptavidin.

### Cell lines and cell culture

All adherent cell lines were cultured in Dulbecco’s Modified Eagle Medium (DMEM, Gibco, Massachusetts, Waltham), 1% Pen/Strep (Gibco), 10% Fetal Calf Serum (FCS, Biowest, France, Nuaillé). The U266 cells were cultured in IMDM (Lonza, Switzerland, Basel), 10% FCS (Gibco, USA, Massachusetts, Waltham), 3 mM L-glutamine (Lonza, Switzerland, Basel), 1% Pen/Strep. HeLa cells were transduced with the aid of a pEF1α lentiviral vector encoding the cDNA of HLA-A2 or GFP as a Mock. In the HeLa-HLA-A2 (HeLa-A2) cell line EGFR and Her2 knockout lines were generated as described before [22]. The U266 cell line and one of the HeLa-A2 knockout Her2 clones were transduced with MP71 retroviral vectors encoding an intracellular domain truncated Her2 or EGFR receptor (tHer2 or tEGFR). Cell cultures were enriched for transduced cells by FACS sorting using an Aria III cell sorter (BD Bioscience, Germany, Heidelberg).

The T-cells used were specific for EBV-BRLF1 (YVLDHLIVV presented in HLA-A*02:01), or CMV-pp65 as a non-specific control (NLVPMVATV presented in HLA-A*02:01) and were either previously established T-cell clones or CD8^+^ T-cells derived from peripheral blood mononuclear cells (PBMCs) transduced with the virus-specific TCRs. T-cells were cultured in T-cell medium (TCM); IMDM (Lonza, Switzerland, Basel), 5% FBS (Gibco, USA, Massachusetts, Waltham), 5% human serum (Sanquin, the Netherlands, Amsterdam), 3 mM L-glutamine (Lonza, Switzerland, Basel), 1% Pen/Strep, and 200 IU/ml IL-2, and stimulated every 10-16 days with phytohaemagglutinin (PHA, ThermoFisher, Germany, Dreieich) and irradiated feeder cells. Before the T-cells were used in the assays cells were washed 3 times with IMDM supplemented with 0.5% human albumin (Albuman, Sanquin) to remove expansion-related cytokines. Mycoplasma tests were performed regularly by PCR and were negative throughout the duration of the experiments.

### TCR identification and TCR gene transfer to CD8^+^ T-cells

TCRs of selected T-cell clones were sequenced [27]. The TCR chains were codon optimized, synthesized, and cloned in a MP71-TCR flex retroviral vector by Baseclear (the Netherlands, Leiden). The MP71 flex retroviral vector contains codon-optimized and cysteine-modified TCRαβ (mTCR) constant domains to optimize TCR expression and increase preferential pairing [28]. Phoenix-AMPHO cells were transiently transfected with the generated constructs and virus supernatant was harvested and stored at −80 °C. From PBMCs, CD8^+^ T-cells were isolated by Magnetic-activated cell sorting (MACS) with CD8^+^ isolation beads (Miltenyi Biotec) and stimulated with PHA with irradiated autologous feeder cells at a 1:3 ratio. On day 2 after stimulation, 24 well suspension plates were coated with retronectine (Takara, France, Paris) and blocked with 2% human serum albumin (HSA). Next, the retroviral supernatant was added, and cells were spun down for 20 min at 4 °C, 2000 × *g*. The retroviral supernatant was removed, and the activated T-cells were transferred to the wells. After overnight incubation, the T-cells were transferred to a new culture plate. On day 7 after stimulation the TCR positive cells were MACS enriched using an anti-mouse TCR-Cβ-APC (mTCR, BD Pharmingen, Germany, Heidelberg) antibody in combination with anti-APC beads (Miltenyi Biotec). On day 8–10 after stimulation the T-cells were analyzed on a FACS for CD8, and mTCR expression, and transduced T-cells were used in experiments or re-stimulated to expand further.

### Flow cytometry

For flow cytometry experiments, 100,000 cells were plated in a 96-wells U-bottom plate, washed with PBS containing 0.5% bovine serum albumin (BSA) and 0.02% Natrium Azide (PBA), and incubated with primary antibody for 30 min on ice. Hereafter, the cells were washed 2x with PBA, followed with 20 min incubation with the secondary antibody. The cells were washed once and analyzed on a LSRII (BD, USA, New Jersey, Franklin Lakes). The amount of cell surface receptors was quantified for the different cell lines, using the QIFI kit (Agilent Dako, USA, Santa Clara) according to the manufacturer’s instructions.

### T-cell activation assays

For the T-cell activation assays with adherent cell lines, 5000 target cells/well were cultured overnight in a 384-well flat-bottom tissue culture plate to allow them to adhere. Antibody dilutions were prepared in IMDM supplemented with 0.5% HSA and titrated concentration of the different antibodies were added to the adherent cells and incubated for 1 hr at 37 °C. The wells were washed 3x and subsequently 4000 T-cells/well were added to the adherent target cells in IMDM supplemented with 0.5% HSA and 100 IU/ml IL-2. Before the T-cells were added, they were washed 3x to remove expansion-related cytokines. After overnight coculture, IFN-γ production by the T-cells was measured in the supernatant by ELISA as a measure for T-cell activation (Diaclone, France, Besançon).

For the target cell lines in suspension, the tumor cells were exposed to the AECs diluted in IMDM supplemented with 0.5% HSA for 1 hr at 37 °C. Next, cells were washed 3x with IMDM supplemented with 0.5% HSA, and 40,000 cells/well were transferred to a 384-well flat-bottom tissue culture plate to which 4000 T-cells/well were added in IMDM supplemented with 0.5% HSA and 100 IU/ml IL-2.

### T-cell cytotoxicity measurements

After harvesting supernatants from the cocultures of AEC incubated adherent cell lines and T-cells TCM was added and the cocultures were incubated for an additional 48 hrs. Subsequently, T-cells were removed by gentle washing (3x), and DMEM culture medium supplemented with 10% AlamarBlue HS cell viability reagent (ThermoFisher, USA, Massachusetts, Waltham) was added. Viability was measured in relative fluorescence units (RFU) according to the manufacturer’s protocol. The percentage target cell killing was calculated using:

$${\%} {\,}{\rm{killing}}=100-\frac{({\rm{RFU}}\,{\rm{sample}}-{\rm{average}}\,{\rm{RFU}}\,{\rm{background}})}{({\rm{RFU}}\,{\rm{no}}\,{\rm{addition}}-{\rm{average}}\,{\rm{RFU}}\,{\rm{background}})} {\times} 100$$

In which RFU sample is the measured value of our samples, the average RFU background is the average value that comes from four wells in which only T-cells were cultured, and RFU no addition is the measured value of a coculture of target and T-cells without antibody or peptide treatment.

To measure T-cell-mediated cytotoxicity of the different U266 cells, the U266-tEGFR and U266-tHer2 were incubated for 1 hr with 100 nM of AEC at 37 °C. After removal of the AEC by 3 centrifugation steps with IMDM with 0.5% HSA, the U266 cells were cultured at 37 °C for 18 hr. Then the U266 cells were incubated 1 hr at 37 °C with 100 μCi ^51^chromium (PerkinElmer, USA, Massachusetts, Waltham), washed 3 times, and incubated at different E:T ratios for 7 h in 96-well U-bottom culture plates. The spontaneous release was measured by addition of only IMDM with 0.5% HSA and the maximum release by addition of 1% Triton X-100 (Sigma–Aldrich, USA, Missouri, Saint Louis). The supernatants were harvested and transferred to LumaPlates (PerkinElmer) and ^51^Cr release was measured on a 2450 Microbeta2 plate counter (PerkinElmer) in counts per minute (CPM). The percentage killing was calculated with a similar formula as for the Alamarblue assay.

### In vivo experiments

Animal procedures were performed according to AVD116002017891 appendix 2 which was approved by the Central Committee of Animal Experiments (CCD, The Hague, The Netherlands) according to the European legislation (EU 2010/63/EU) and Animal Experimental Committee of Leiden University.

Female NOD-scid-IL2Rgammanul (NSG) 7–14 weeks old mice were injected intravenously (i.v.) with 2 × 10^6^ U266 cells transduced with the extracellular domain of Her2 (tHer2) and Luciferase. After 14 days, the mice were injected i.v. with 5 × 10^6^ T-cells transduced with the virus-specific TCR, followed by an i.v. injection on day 15 and 18 of the TRS-H (100 μg) diluted in PBS or an intraperitoneal (i.p.) injection of 100 μg on day 14 and 18 of CTX-H as a single treatment or in combination with 100 μg pembrolizumab. Tumor outgrowth was measured at regular intervals after subcutaneous (s.c.) injection of 150 μL 7.5 mM d-luciferine (Cayman Chemical) using a CCD camera (IVIS Spectrum, PerkinElmer). On the indicated days, blood was collected through a tail-vein puncture, and serum was collected and frozen. Group sizes were determined bases on variation observed in previous experiments and mice were randomized over groups based on the tumor outgrowth as measured on day 14. Treatment was not blinded.

### Statistical analysis

Graphpad Prism software (V.9.0.1) was used to perform the statistical analysis. In the figure legend the used test is indicated, and the significance levels are indicated as ns as not significant, *p < 0.05, **p < 0.01, ***p < 0.001, and ****p < 0.0001.

## Results

### EBV epitope selection

**Fig. 1:**
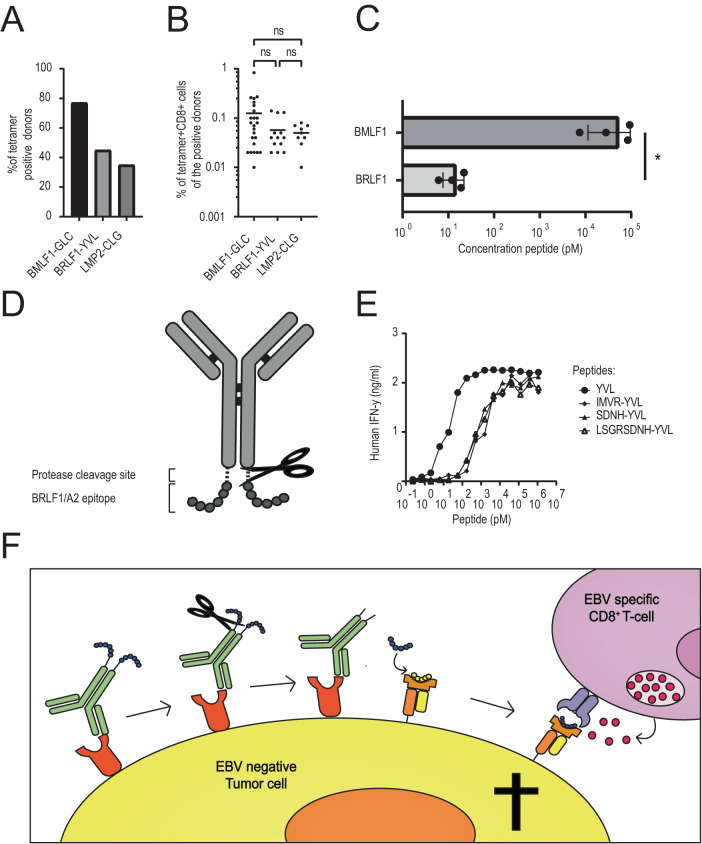
N-terminally extended T-cell epitopes can efficiently be presented by cancer cells and antibodies can be genetically modified to contain these epitopes at their C-terminus. **A** PBMCs of 33 HLA-A2+ healthy donors that were EBV seropositive were stained with EBV tetramers and analyzed by FACS. Depicted are the percentage of donors that showed EBV tetramer positive CD8 T-cells ( > 0.01%). The three EBV tetramers that were recognized most often are depicted. **B** Depicted are the percentage of CD8^+^ T-cells within the EBV-seropositive HLA-A2 positive donors that demonstrated EBV tetramer positivity. Each dot represents the measurement of 1 donor. For the statistical analyses, a one-way ANOVA was used with Tukey’s multiple comparisons. **C** MDA-MB231 cells were pulsed with either the BRLF1-YVL or the BMLF1-GLC epitope, which were titrated to determine the half maximal effective concentration (EC_50_). The pulsed target cells were cocultured for 18 hrs with at least 3 different epitope-specific T-cell clones in two independent experiments. T-cell activation was analyzed by measuring IFN-γ production of the T cells in the supernatant. Plotted values are the EC_50_ values calculated from the means of duplicates (SEM). To determine statistical significance, an unpaired *T*-test was performed. **D** The AEC concept in which the epitope, preceded by a protease cleavage site, is attached to the C-terminus of the antibody. **E** MDA-MB231 cells were pulsed for 1 hr with titrated concentrations of the YVL epitope containing either no (YVL), the natural (IMVR-YVL) or (part of) a protease cleavage site (SDNH-YVL or LSGRSDNH-YVL), followed by coculture with BRLF1-YVL specific T-cells for 18 hrs, after which IFN-γ production by the T cells was measured in the supernatant. **F** Schematic overview of the mechanism of the concept, in which the antibody first recognized the antibody target, followed by proteolytic release of the epitope by proteases secreted by the tumor cell. After release the epitope eventually gets presented on the HLA molecules present on the tumor cells which can get recognized by the virus-specific CD8^+^ T-cell.

To select the most suitable EBV epitope for conjugation to the tumor-specific antibodies, the prevalence of different EBV-specific T-cells in a cohort of healthy donors was determined. Peripheral blood mononuclear cells (PBMCs) of HLA-A*02:01-positive and EBV-seropositive donors (HLA-A2^+^/EBV^+^) were stained with peptide major histocompatibility complex (pMHC) tetramers representing several different HLA-A2 restricted EBV epitopes [11]. Within the HLA-A2^+^/EBV^+^ analyzed donors (*n* = 31), three epitopes were most frequently recognized: BMLF1-GLC, BRLF1-YVL, and LMP2-CLG (Fig. 1A), of which the BMLF1-GLC epitope was recognized most often (77%), followed by the BRLF1-YVL epitope (45%). The percentage of CD8^+^ T-cells able to recognize the BMLF1-GLC and BRLF1-YVL pMHC tetramers varied between 0.05–0.13%, with no significant difference between these two epitopes (Fig. 1B).

To determine the potency of the two EBV-specific T-cells, MDA-MB231 cells were pulsed with the titrated, cognate peptide and cocultured with BMLF1-GLC or BRLF1-YVL specific CD8^+^ T-cells. Interferon-γ (IFN-γ) concentrations in the culture supernatants were determined as a measure for T-cell activation and the half maximal effective concentration (EC_50_ value) of the titrated peptide was calculated. Although a larger fraction of HLA-A2^+^/EBV^+^ donors recognized the BMLF1-GLC epitope, BRLF1-YVL CD8^+^ T-cells were a 100-fold more potent in recognizing target cells exogenously loaded with the BRLF1-YVL peptide compared to the BMLF-GLC specific T-cells (Fig. 1C). Therefore, the BRLF1-YVL epitope was selected to use for the AEC strategy.

### An N-terminally extended BRLF1-YVL epitope can efficiently activate EBV-specific T-cells

The release mechanism of the antibody-epitope conjugates (AECs) concept relies on the release of the epitope by proteases present in the extracellular environment (Fig. 1D). To determine whether epitopes elongated with a protease cleavage site on the N-terminus could be cleaved of the antibodies and eventually be presented by the tumor cells, MDA-MB231 cells were first exposed to different N-terminal extended peptides. The N-terminal extensions consisted of the natural extension of the BRLF1-YVL epitope in EBV (IMVR-), a cleavage site (LSGRSDNH-) specific for the proteases urokinase-type plasminogen activator (uPA), membrane-type serine protease 1 (MT-SP1/matriptase), and legumain [29], or the 4 amino acids of this cleavage site that may remain after protease cleavage (SDNH-). The T-cell activation observed for all N-terminally extended peptides was 10-fold lower compared to MDA-MB231 cells pulsed with the minimal epitope, however, the half maximal effective concentration (EC_50_ value) of the extended peptides were still in picomolar range (Fig. 1E). These data demonstrate that an N-terminally extended BRLF1-YVL epitope can be exogenously loaded on the HLA molecules of tumor cells, resulting in specific T-cell activation.

### Virus-epitopes can be efficiently linked to the C-terminus of the heavy chain of tumor-specific antibodies

Next, the N-terminally extended BRLF1-YVL epitope was genetically linked to the heavy chain (HC), the light chain (LC), or both chains ((AECs-H, -L, or HL) of trastuzumab (TRS) and cetuximab (CTX)), to function as a delivery vehicle for the BRLF1-YVL epitope preceded by the protease cleavage site (Fig. 1F). The presence of the epitopes on the antibodies was analyzed with SDS-Page (Fig. S1A, B) and with LC–MS it was confirmed that in almost all (>90%) antibodies the epitope was intact (Fig. S1C). This demonstrates that genetically fused AECs with an epitope-to-antibody ratio (EAR) of 2 or 4 were generated.

**Fig. 2:**
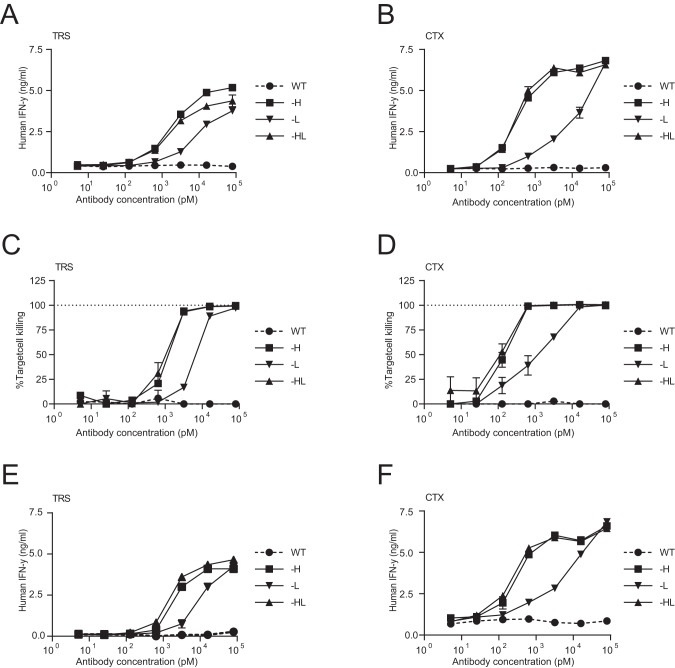
Tumor cells treated with AECs activate virus-specific T cells. **A**, **C** HeLa-A2 tHer2 cells were pulsed for 1 hr with titrated concentrations of wildtype trastuzumab (WT) or TRS-H, TRS-L or TRS-HL, and cocultured with EBV-specific T-cells. **B**, **D** HeLa-A2 cells were pulsed with titrated concentrations of wildtype cetuximab (WT) or the AECs CTX-H, CTX-L, or CTX-HL, and cocultured with EBV-specific T-cells. **A**, **C** T-cell activation after overnight coculture was analyzed by measuring IFN-γ production of the T cells in the supernatant. **B**, **D** To measure target cell killing, an AlamarBlue assay was performed, in which the AEC incubated HeLa-A2 cells were cocultured for 72 hrs with the virus-specific T-cells. **E**, **F** To determine whether we could also achieve T-cell activation in a more primary T-cell setting, CD8^+^ T-cells were isolated from healthy donors and transduced with an BRLF1-YVL-specific TCR. The T-cell activation assays were repeated for **E** all AECs of TRS, and **F** for all AECs of CTX. Plotted values are the means of duplicates (SEM) and each graph shows a representative figure of more than three different experiments.

TRS recognizes the human epidermal growth factor receptor 2 (Her2) and CTX the epidermal growth factor receptor (EGFR). Her2 and EGFR are known to be overexpressed on many cancer cells and both antibodies are successful clinical strategies. To compare the efficiency of the different TRS- and CTX-AECs, EGFR-expressing HeLa cells transduced with HLA-A2 (HeLa-A2) and truncated Her2 (HeLa-A2 tHer2) were exposed to the different AECs and subsequently cocultured with the BRLF1-YVL specific CD8^+^ T-cells. All TRS- and CTX-AECs resulted in T-cell activation (Fig. 2A, B), however, the potency of T-cell activation of TRS-L and CTX-L AECs was lowered compared to -H and -HL AECs. In addition, no increase in T-cell activation for the -HL AECs compared to the -H AECs was found, indicating that an additional epitope on the LC did not increase T-cell activation. This was also observed when target cell killing was measured (Fig. 2C, D). These results suggest that the BRLF1-YVL epitope on the LC is not released or loaded into HLA-A2 as efficiently as the epitope on the HC.

To investigate whether the different AECs showed similar activation of virus-specific T-cells when incubated with other tumor cell lines expressing the endogenous tumor antigens, we repeated the experiments with the tumor cell lines SKOV3 (ovarium carcinoma) and H292 (lung cancer) transduced with HLA-A2 (SKOV3-A2 and H292-A2) and MDA-MB231 (breast cancer) which expresses HLA-A2 endogenously. All three tumor cell lines express high levels of EGFR and SKOV3-A2 cells also expresses high levels of Her2 (Fig. S2A). Similar to previous results with HeLa-A2, the TRS- and CTX-AECs -H and -HL showed activation of the BRLF1 specific T-cells when incubated with different target antigen positive tumor cell lines. Moreover, the ability of TRS-L and CTX-L AECs to induce T-cell activation was again in all cases less efficient than -H or -HL AECs (Fig. S2B). In conclusion, increasing the EAR of AECs by adding an additional epitope to the C-terminus of the LC did not improve the efficiency of epitope delivery and concomitant T-cell activation.

### Virus-specific T-cells are activated by AECs in a target-specific manner

**Fig. 3:**
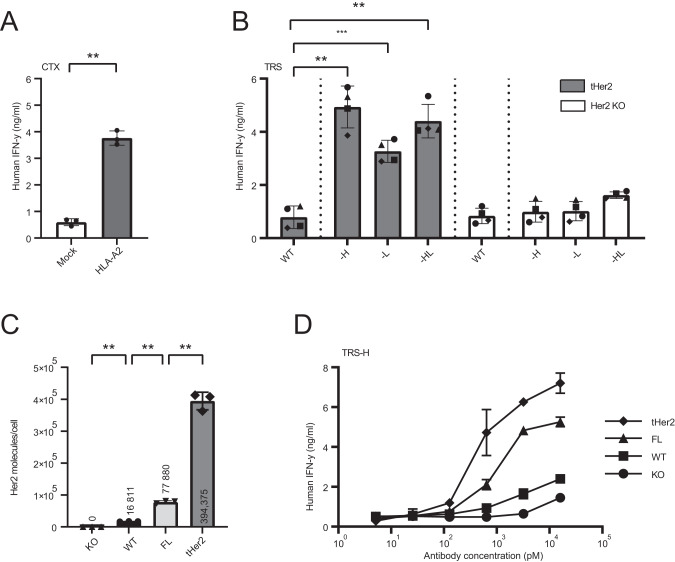
HLA-A2 and higher expression levels of antibody target are required for an efficient T-cell response. **A** To investigate the HLA-A2 necessity on the tumor cells, HeLa cells transduced with HLA-A2 or GFP (Mock) were incubated with AEC CTX-H (16 nM), and cocultured with EBV T-cells overnight. T-cell activation was analyzed by measuring IFN-γ in the supernatant. To determine statistical significance, a paired T-test was performed. **B** To investigate the antibody target necessity, HeLa-A2 tHer2 and HeLa-A2 Her2 KO cell lines were incubated with all variants of the AEC TRS (16 nM) and cocultured with EBV T cells overnight. To determine statistical significance, a RM one-way ANOVA with Tukey’s multiple comparisons was performed. **C** The amount of Her2 molecules expressed on the cell membrane of the 4 different HeLa-A2 were quantified with the QIFI kit. Cells used are HeLa-A2 cells with Her2 knockout (KO), HeLa-A2 with wildtype Her2 (WT) expression levels, HeLa-2A KO transduced with full-length Her2 (FL) or lacking the intracellular domain of Her2 (tHer2). **D** The effect of different Her2 expression levels was analyzed in a coculture assay in which EBV T-cells were cocultured overnight with the different HeLa-A2 cells incubated with titrated concentrations of the AEC TRS-H. T-cell activation was analyzed by measuring IFN-γ in the supernatant. **A**, **B**, **D** T-cell activation was analyzed by IFN-γ ELISA. **A**, **B** Plotted values are the means of duplicates (SEM) of 3–4 independent experiments. **D** Graph shows a representative figure of three different experiments.

In the previous functional assays BRLF1-YVL specific CD8^+^ T-cell clones isolated from EBV-seropositive donors were used. To validate these results with primary CD8^+^ T-cells, the TCR of the BRLF1-YVL specific T-cell clone was sequenced and a retroviral vector encoding the TCR was generated. The retroviral vector was used to transfer the BRLF1 TCR DNA into primary CD8^+^ T-cells isolated from healthy donors (Fig. S3). The AEC-exposed HeLa-A2 cell lines were cocultured with the BRLF1 TCR T-cells and IFN-γ production was determined as a measure of T-cell activation. Similar results were obtained with the EBV-TCR T-cells compared to the EBV-specific T-cell clones (Fig. 2E, F) and T-cell activation was not observed when the CD8^+^ T-cells were transduced with a non-specific TCR (Fig. S4) In addition, since the healthy donors used were all HLA-A2-negative these results also indicated that the released EBV epitopes were presented only by the HLA-A2 on the HeLa-A2 cells. This did not exclude the possibility of epitopes being released by the AECs that could eventually be presented on HLA class I molecules of neighboring cells, which could potentially lead to destruction of healthy cells. To rule out presentation of the AEC-released epitopes on neighboring cells, we transduced HeLa cells either with GFP (Mock) or HLA-A2 and pulsed them with CTX-H followed by coculture of the BRLF1-YVL specific CD8^+^ T-cell clone, which was HLA-A2 positive. Only when target-positive tumor cells expressed HLA-A2, T-cell activation was observed (Fig. 3A). These results demonstrate that HLA-A2 expression on the tumor cell lines themselves is required and that after protease cleavage the epitope is not presented by an HLA-A2^+^ T-cell in close vicinity of the target-positive tumor cells.

In addition, we investigated whether targeting tumor cells with the different AECs was target antigen-specific. Coculture of the EBV-specific T-cells was performed with AEC-H, -L, and -HL exposed HeLa-A2 tHer and HeLa-A2 Her2 KO cell lines (Figs. 3B and S5A). Significant T-cell activation was observed for the three AECs when Her2 was present on the HeLa cells. From these data we conclude that both HLA-A2 and the antibody target are necessary on the tumor cells for epitope presentation and T-cell activation.

### High expression levels of the antibody target are required to induce T-cell activation

Since antibody target expression may influence epitope delivery, we investigated with four HeLa-A2 cell lines expressing varying Her2 levels the sensitivity for AEC-mediated T-cell recognition. Flow cytometry using a Her2 specific antibody revealed the variation in expression (Fig. S5B), and Her2 expression per cell was quantified using the QIFI kit (Fig. 3C). In addition, the number of HLA-A2 molecules per cell were quantified and demonstrated to be similar among all 4 HeLa cell lines (Fig. S5C, D). The different HeLa cell lines were exposed to TRS-H, followed by a coculture with the BRLF1-YVL specific T-cells. As shown in Fig. 3D, the amount of Her2 correlated with the level of T-cell activation, and the data illustrated that high target levels (>70,000 Her2 molecules/cell) are necessary to obtain high levels of T-cell activation.

### AEC-redirected EBV-specific T-cells exert potent tumor reduction in vivo

**Fig. 4:**
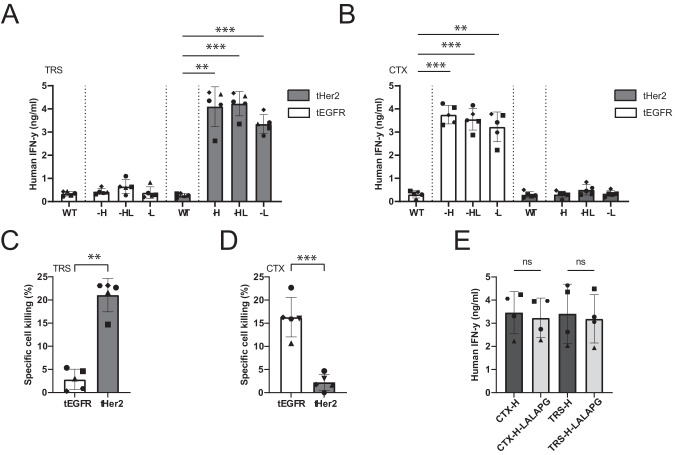
U266 cells treated with AECs activate virus-specific T cells. **A**, **B** U266-tHer2 or -tEGFR were incubated with 10 nM of the different AECs of **A** TRS or **B** CTX, and cocultured with EBV-specific T cells overnight. T-cell activation was analyzed by measuring IFN-γ in the supernatant. Plotted values are the means of duplicates (SEM) of 4 independent assays with either the BRLF1 T-cell clone or BRLF1 TCR T-cells. **C**, **D** To investigate whether the U266-tHer2 or -tEGFR upon AEC incubation were also killed by the EBV T cells killing assays were performed. U266-tHer2 and -tEGFR were incubated for 1 hr with 100 nM of the AEC **C** TRS-H or **D** CTX-H, washed, cultured for an addition day, and used in cytotoxicity assays. Killing was measured by ^51^Cr release assay after 7 hrs of coculture. Three independent assays were performed with T-cell clones or isolated CD8^+^ T-cells transduced with the EBV-specific TCR. Plotted values are the means of technical triplicates. To determine statistical significance, a paired T-test was performed. **E** Comparison of the functional activity of Fc-inert CTX (on U266-tEGFR) and TRS (on U266-tHer2) with the non-inert versions on U266 target-positive cells (16 nM), and subsequent coculture with EBV-specific T cells overnight. T-cell activation was analyzed by IFN-γ ELISA and plotted values are the means of duplicates in 4 independent assays with either the BRLF1 T-cell clone or BRLF1 TCR T-cells.

To determine the effectiveness of our AEC approach in vivo, we transduced the multiple myeloma (MM) cell line U266 with retroviral vectors encoding truncated Her2 or EGFR, lacking the intracellular signaling domains (U266-tHer and U266-tEGFR) to rule out unwanted effects of Her2 or EGFR signaling. Transduced U266 cells were efficiently recognized by EBV-specific T-cells upon incubation with their specific AECs. (Fig. 4A, B). In addition, the AEC-redirected EBV-specific T-cells efficiently killed the transduced U266 cells in a target-specific manner (Fig. 4C, D). These results again support the target specificity of the AECs and their ability to redirect the virus-specific T-cells towards tumor cells, resulting in activation of T-cells and subsequently killing of the tumor cells.

**Fig. 5:**
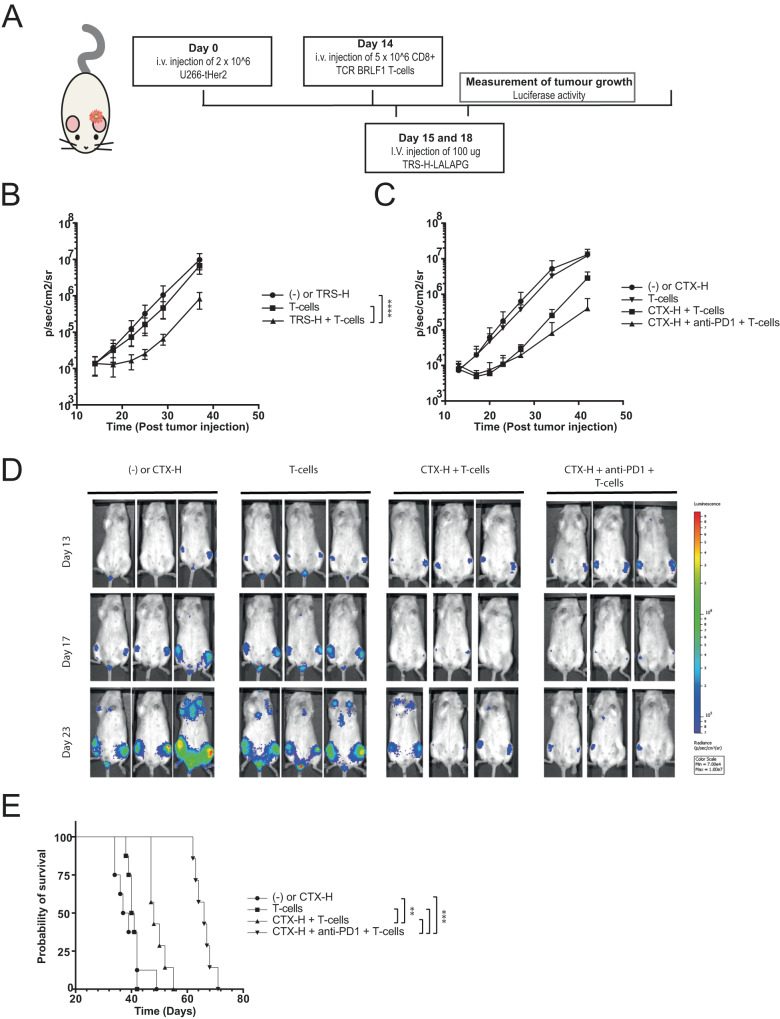
AECs reduce tumor outgrowth by redirecting EBV-specific T-cells in vivo. **A** Overview of the experimental set-up of the in vivo experiment of **B** NSG mice engrafted with 2 × 10^6^ U266-tHer2 cells transduced with luciferase were i.v. injected with 5 × 10^6^ EBV-TCR-transduced CD8 T-cells at day 14. On day 15 and 18 mice were i.v. injected with 100 g TRS-H. Tumor outgrowth of the U266-tHer2 was visualized by bioluminescence imaging 1-2 times per week of the ventral side. Significance was determined with a two-way ANOVA, with a Tukey’s multiple comparisons. **C** NSG mice engrafted with 2 × 10^6^ U266-tEGFR were i.v. injected at day 14 with 5 × 10^6^ EBV-TCR-transduced CD8 T-cells. On day 14 and 17 mice were intraperitoneal (i.p) injected with 100 μg CTX-H alone or in combination with 100 μg pembrolizumab. **D** Representative images at days 13, 17, and 23 of U266 tumor burden as measured on the IVIS. **E** A Kaplan–Meier plot was created with the different groups in which also the significance was calculated. The endpoint was determined when the tumor outgrowth reached a bioluminescent signal of 1 × 10^7^ p/s/cm^2^/sr.

For the in vivo experiments, TRS-H and CTX-H AECs were produced with the L234A, L234A, and P39G (LALAPG) mutations, to prevent killing through antibody-dependent cell-mediated cytotoxicity (ADCC), phagocytosis or complement activation and to study the sole effect of the antibody-mediated redirection of virus-specific T-cells (Fig. S6) [30]. The EGFR and Her2 specific AECs with the LALAPG mutations were evaluated in functional assays and demonstrated similar potency compared to the AECs with the wt IgG1 Fc domain (Figs. 4E, S7). Next, NSG mice were injected with luciferase-expressing U266-tHer2 cells, which will engraft into the bone marrow. On day 14 after tumor engraftment EBV-TCR T-cells were infused prior to AEC treatment on days 15 and 18 to allow the T-cells to engraft first (Fig. 5A). Immediately after the first AEC injection, tumor outgrowth was significantly reduced in mice treated with T-cells and AEC compared to control mice that only received T-cells or AEC (Fig. 5B).

To assess whether the AEC upon infusion into the mice still contained intact epitopes, serum was collected from the mice at day 21 after tumor engraftment (3 days after last AEC infusion). HeLa-A2 tHer2 and Her2 KO cells were incubated with the collected sera followed by flow cytometry or cocultured with the BRLF1-YVL specific T-cells (Fig. S8A and B). Results show that HeLa tHer2 cells preincubated with serum from mice treated with TRS-H AEC were able to efficiently activate the EBV epitope-specific T-cells, indicating that 3 days after i.v. injection, the AEC TRS-H was still intact and present in the blood of mice treated with the AEC.

### Additional immune checkpoint blockade improves long-term immune control in vivo

As the AEC was not the limiting factor on day 21 when the tumor started to grow out, possible explanations for the reduced effect on tumor outgrowth could be T-cell exhaustion or saturation of the antibody target. When exhaustion would occur, the treated mice could benefit from the addition of immune checkpoint blockade (ICB). To investigate this, NSG mice engrafted with U266-tHer2 were infused with EBV-TCR T-cells on day 14 after which the mice were treated with AEC CTX-H alone or with AEC CTX-H in combination with pembrolizumab, a monoclonal antibody directed against programmed cell death receptor 1 (PD-1). Both CTX-H treatment groups showed a clear reduction in tumor size compared to mice treated with T-cells or AEC only (Fig. 5C, D). The additional effect of pembrolizumab on the T-cells was not observed when CTX-H was not administered (Fig. S9B). Interestingly, two weeks after the last dose administered, mice that received pembrolizumab showed a slower tumor outgrowth, eventually resulting in a significantly prolonged survival (Fig. 5E). These results demonstrate that treatment with AECs positively benefit from ICB as a combination therapy.

## Discussion and conclusion

Earlier studies showed that AECs generated with chemical conjugation strategies [18, 19] are a promising strategy to deliver immunogenic viral epitopes and redirect virus-specific CD8^+^ T-cells towards cancer. Here, we show the feasibility of this approach with two AECs with genetically fused viral epitopes both in vitro and in vivo. We demonstrate that AECs with an EBV epitope attached to each HC most potently redirect EBV-specific T-cells towards multiple tumor cell lines, and that the approach is target-specific and potency correlates with target expression. In addition, the AECs redirected virus-specific T-cells in vivo, which led to reduction in tumor outgrowth, and prolonged survival of mice. Taken together, we conclude that genetically fused AECs can efficiently redirect and activate virus-specific T-cells by delivering viral epitopes for anti-cancer therapy.

The design of the AECs-H with the EBV-BRLF1 epitope proved to be highly efficient in delivering the viral epitopes to multiple cancer cell lines and further optimization could most likely even extend and improve the design further. The efficiency of epitope delivery of AEC-Ls was lower compared to AEC-Hs (Figs. 2 and S2), which could be caused by a more shielded protease cleavage site, rendering it less accessible. Improving the accessibility could potentially give rise to an increased EAR, and also the possibility to attach two different viral epitopes to one AEC. Secondly, further testing and selection of other antibodies or antibody targets, with different affinities and optimal receptor/AEC turnover time could also potentially further improve the strategy. CTX and TRS are known to remain on the cell surface as antibody-receptor complexes for long periods of time, preventing ligand binding and dimerization of the EGF receptors [31, 32], resulting in restricted opportunities for non-bound AECs to recognize and deliver new viral epitopes. This could explain the limited effect after the initial tumor reduction observed in vivo (Fig. 5).

Based on presented results and as further explained hereafter, we hypothesize that AECs exhibit a higher safety profile compared to other antibody therapies, such as the CD3-BsAbs, or CAR T-cells. We demonstrate that the antibody target expression levels and therefore the level of antibody target recognition on tumor cells influences the presentation of viral epitopes (Fig. 3). Low levels of target expression did not result in T-cell activation. In contrast, several monoclonal antibodies and CAR T-cells targeting antigens on solid tumors also demonstrate recognition of healthy tissues, and this has been a major hurdle for the use of therapeutic strategies that aim at redirecting all CD3^+^ T-cells [7, 8, 33]. In addition, the protease cleavage site that is placed between epitope and antibody provides an additional safety mechanism of AECs. It is known that cancer cells overexpress multiple proteases, which are secreted into the tumor microenvironment (TME) [34–36]. This reduces the chance of release of the viral epitope in healthy tissues and allows for administration of higher doses, which has already been shown to be effective for prodrugs [37] and probodies [29, 38, 39]. Moreover, with the AEC approach a smaller fraction of T-cells will be redirected, which most likely will decrease the chances of adverse effects like cytokine-release syndrome.

As a smaller group of CD8^+^ T-cells is targeted by AECs, a potential limitation could be the absence of these epitope-specific T-cells in the TME. The epitope can be delivered to the tumor cells, however, when the epitope-specific T-cells are not present there will be no virus-specific T-cell activation. In these cases, adoptive T-cell transfer and introduction of virus-specific TCRs in the patient’s own CD8^+^ T-cells could be a solution, as has been demonstrated previously in patients after transplantation [40, 41].

Next to the absence of these T-cells in the TME, heterogeneity of TAA expression or downregulation of HLA on the tumor cells could be a potential limitation. More recent publication reported intratumoral injection of viral epitopes and subsequent activation of virus-specific T-cells within the TME boosted long-term anti-tumor immune responses [16, 42], which may indicate that triggering of long-term anti-tumor immune responses with our AECs may also occur. In general, AECs have mainly been evaluated for their capability to redirect and activate CD8^+^ T-cells in vitro and in vivo [18, 19]. In these experiments long-term anti-tumor responses, but also additional antibody effector functions, such as ADCC or complement-driven cytotoxicity (CDC) [43] were not studied and could help with overcoming these potential limitations. Moreover, intratumoral injection of viral epitopes may lead to killing of healthy cells, within and around the tumor, also presenting these viral epitopes. Based on our results demonstrating that after protease cleavage the epitopes were only presented by target-positive tumor cells (Fig. 4), we anticipate that AECs will not exhibit these safety issues.

We conclude that genetically fused AECs can effectively redirect virus-specific T-cells. We demonstrated that the genetically fused AECs can significantly reduce tumor outgrowth by redirecting the virus-specific CD8^+^ T-cells in vivo (Fig. 5). This makes this approach an exciting therapeutic avenue to be further explored to move new treatment modalities forward towards clinical evaluation.

### Supplementary information

Supplemental material

## Data Availability

All data are available in the main text or the supplementary materials.

## References

[1] Przepiorka D, Ko CW, Deisseroth A, Yancey CL, Candau-Chacon R, Chiu HJ (2015). FDA approval: blinatumomab. Clin Cancer Res.

[2] Schuster SJ, Bartlett NL, Assouline S, Yoon SS, Bosch F, Sehn LH (2019). Mosunetuzumab induces complete remissions in poor prognosis non-hodgkin lymphoma patients, including those who are resistant to or relapsing after chimeric antigen receptor T-Cell (CAR-T) therapies, and is active in treatment through multiple lines. Blood.

[3] Middleton MR, McAlpine C, Woodcock VK, Corrie P, Infante JR, Steven NM (2020). Tebentafusp, a TCR/Anti-CD3 bispecific fusion protein targeting gp100, potently activated antitumor immune responses in patients with metastatic melanoma. Clin Cancer Res.

[4] Middelburg J, Kemper K, Engelberts P, Labrijn AF, Schuurman J, van Hall T (2021). Overcoming challenges for CD3-bispecific antibody therapy in solid tumors. Cancers.

[5] Clynes RA, Desjarlais JR (2019). Redirected T cell cytotoxicity in cancer therapy. Annu Rev Med.

[6] Thommen DS, Schumacher TN (2018). T cell dysfunction in cancer. Cancer Cell.

[7] Lutterbuese R, Raum T, Kischel R, Hoffmann P, Mangold S, Rattel B (2010). T cell-engaging BiTE antibodies specific for EGFR potently eliminate KRAS- and BRAF-mutated colorectal cancer cells. Proc Natl Acad Sci USA.

[8] Ellerman D (2019). Bispecific T-cell engagers: towards understanding variables influencing the in vitro potency and tumor selectivity and their modulation to enhance their efficacy and safety. Methods.

[9] Teachey DT, Rheingold SR, Maude SL, Zugmaier G, Barrett DM, Seif AE (2013). Cytokine release syndrome after blinatumomab treatment related to abnormal macrophage activation and ameliorated with cytokine-directed therapy. Blood.

[10] Klenerman P, Oxenius A (2016). T cell responses to cytomegalovirus. Nat Rev Immunol.

[11] Hislop AD, Taylor GS, Sauce D, Rickinson AB (2007). Cellular responses to viral infection in humans: lessons from Epstein-Barr virus. Annu Rev Immunol.

[12] Cicin-Sain L, Arens R (2018). Exhaustion and inflation at antipodes of T cell responses to chronic virus infection. Trends Microbiol.

[13] Yewdell JW, Bennink JR (1999). Immunodominance in major histocompatibility complex class I-restricted T lymphocyte responses. Annu Rev Immunol.

[14] Bentzen AK, Marquard AM, Lyngaa R, Saini SK, Ramskov S, Donia M (2016). Large-scale detection of antigen-specific T cells using peptide-MHC-I multimers labeled with DNA barcodes. Nat Biotechnol.

[15] Scheper W, Kelderman S, Fanchi LF, Linnemann C, Bendle G, de Rooij MAJ (2019). Low and variable tumor reactivity of the intratumoral TCR repertoire in human cancers. Nat Med.

[16] Cuburu N, Bialkowski L, Pontejo SM, Sethi SK, Bell ATF, Kim R (2022). Harnessing anti-cytomegalovirus immunity for local immunotherapy against solid tumors. Proc Natl Acad Sci USA.

[17] Jung K, Son MJ, Lee SY, Kim JA, Ko DH, Yoo S (2022). Antibody-mediated delivery of a viral MHC-I epitope into the cytosol of target tumor cells repurposes virus-specific CD8(+) T cells for cancer immunotherapy. Mol cancer.

[18] Millar DG, Ramjiawan RR, Kawaguchi K, Gupta N, Chen J, Zhang S (2020). Antibody-mediated delivery of viral epitopes to tumors harnesses CMV-specific T cells for cancer therapy. Nat Biotechnol.

[19] Sefrin JP, Hillringhaus L, Mundigl O, Mann K, Ziegler-Landesberger D, Seul H (2019). Sensitization of tumors for attack by virus-specific CD8+ T-cells through antibody-mediated delivery of immunogenic T-cell epitopes. Front Immunol.

[20] Erkes DA, Wilski NA, Snyder CM (2017). Intratumoral infection by CMV may change the tumor environment by directly interacting with tumor-associated macrophages to promote cancer immunity. Hum vaccines immunother.

[21] Kang TH, Ma B, Wang C, Wu TC, Hung CF (2013). Targeted coating with antigenic peptide renders tumor cells susceptible to CD8(+) T cell-mediated killing. Mol Ther.

[22] van der Wulp W, Gram AM, Bleijlevens B, Hagedoorn RS, Araman C, Kim RQ (2023). Comparison of methods generating antibody-epitope conjugates for targeting cancer with virus-specific T cells. Front Immunol.

[23] Vink T, Oudshoorn-Dickmann M, Roza M, Reitsma JJ, de Jong RN (2014). A simple, robust and highly efficient transient expression system for producing antibodies. Methods.

[24] Schlothauer T, Herter S, Koller CF, Grau-Richards S, Steinhart V, Spick C (2016). Novel human IgG1 and IgG4 Fc-engineered antibodies with completely abolished immune effector functions. Protein Eng Des Sel.

[25] Brodsky FM, Parham P, Barnstable CJ, Crumpton MJ, Bodmer WF (1979). Monoclonal antibodies for analysis of the HLA system. Immunol Rev.

[26] Burrows SR, Kienzle N, Winterhalter A, Bharadwaj M, Altman JD, Brooks A (2000). Peptide-MHC class I tetrameric complexes display exquisite ligand specificity1. J Immunol.

[27] van Amerongen RA, Hagedoorn RS, Remst DFG, Assendelft DC, van der Steen DM, Wouters AK (2022). WT1-specific TCRs directed against newly identified peptides install antitumor reactivity against acute myeloid leukemia and ovarian carcinoma. J Immunother Cancer.

[28] Linnemann C, Heemskerk B, Kvistborg P, Kluin RJ, Bolotin DA, Chen X (2013). High-throughput identification of antigen-specific TCRs by TCR gene capture. Nat Med.

[29] Desnoyers LR, Vasiljeva O, Richardson JH, Yang A, Menendez EE, Liang TW (2013). Tumor-specific activation of an EGFR-targeting probody enhances therapeutic index. Sci Transl Med.

[30] Overdijk MB, Verploegen S, Buijsse AO, Vink T, Leusen JHW, Bleeker WK (2012). Crosstalk between human IgG isotypes and murine effector cells. J Immunol.

[31] Maass KF, Kulkarni C, Betts AM, Wittrup KD (2016). Determination of cellular processing rates for a trastuzumab-maytansinoid antibody-drug conjugate (ADC) highlights key parameters for ADC design. AAPS J.

[32] Kol A, Terwisscha van Scheltinga A, Pool M, Gerdes C, de Vries E, de Jong S (2017). ADCC responses and blocking of EGFR-mediated signaling and cell growth by combining the anti-EGFR antibodies imgatuzumab and cetuximab in NSCLC cells. Oncotarget.

[33] Morgan RA, Yang JC, Kitano M, Dudley ME, Laurencot CM, Rosenberg SA (2010). Case report of a serious adverse event following the administration of T cells transduced with a chimeric antigen receptor recognizing ERBB2. Mol Ther.

[34] Dall E, Brandstetter H (2016). Structure and function of legumain in health and disease. Biochimie.

[35] Uhland K (2006). Matriptase and its putative role in cancer. Cell Mol Life Sci.

[36] Su SC, Lin CW, Yang WE, Fan WL, Yang SF (2016). The urokinase-type plasminogen activator (uPA) system as a biomarker and therapeutic target in human malignancies. Expert Opin Ther Targets.

[37] Poreba M (2020). Protease-activated prodrugs: strategies, challenges, and future directions. FEBS J.

[38] Wong KR, Menendez E, Craik CS, Kavanaugh WM, Vasiljeva O (2016). In vivo imaging of protease activity by Probody therapeutic activation. Biochimie.

[39] Harmand TJ, Bousbaine D, Chan A, Zhang X, Liu DR, Tam JP (2018). One-pot dual labeling of IgG 1 and preparation of C-to-C fusion proteins through a combination of sortase A and butelase 1. Bioconjug Chem.

[40] Icheva V, Kayser S, Wolff D, Tuve S, Kyzirakos C, Bethge W (2013). Adoptive transfer of epstein-barr virus (EBV) nuclear antigen 1-specific t cells as treatment for EBV reactivation and lymphoproliferative disorders after allogeneic stem-cell transplantation. J Clin Oncol.

[41] Roemhild A, Reinke P (2016). Virus-specific T-cell therapy in solid organ transplantation. Transpl Int.

[42] Rosato PC, Wijeyesinghe S, Stolley JM, Nelson CE, Davis RL, Manlove LS (2019). Virus-specific memory T cells populate tumors and can be repurposed for tumor immunotherapy. Nat Commun.

[43] Wang L, Hoseini SS, Xu H, Ponomarev V, Cheung NK (2019). Silencing Fc domains in T cell-engaging bispecific antibodies improves T-cell trafficking and antitumor potency. Cancer Immunol Res.

