# Supplementary material for: Antibody-mediated delivery of viral epitopes to redirect EBV-specific CD8+ T-cell immunity towards cancer cells
Source: Cancer Gene Ther. 2023 Nov 9;31(1):58–68. doi: 10.1038/s41417-023-00681-4 (PMC10794138; doi:10.1038/s41417-023-00681-4)
Supplement: Supplementary file 1 — Supplemental material [file 41417_2023_681_MOESM1_ESM.pdf]

Supporting information

Antibody-mediated delivery of viral epitopes to redirect EBV-specific CD8+ T-cell immunity towards cancer cells

Willemijn van der Wulp[1], Dennis F. G. Remst[2], Michel G. D. Kester[2], Renate S. Hagedoorn[2], Paul W.H.I. Parren[3], Sander I. van Kasteren[4], Janine Schuurman[5], Rob C. Hoeben[1], Maaike. E. Ressing[1], Boris Bleijlevens[5], Mirjam H. M. Heemskerk[2]

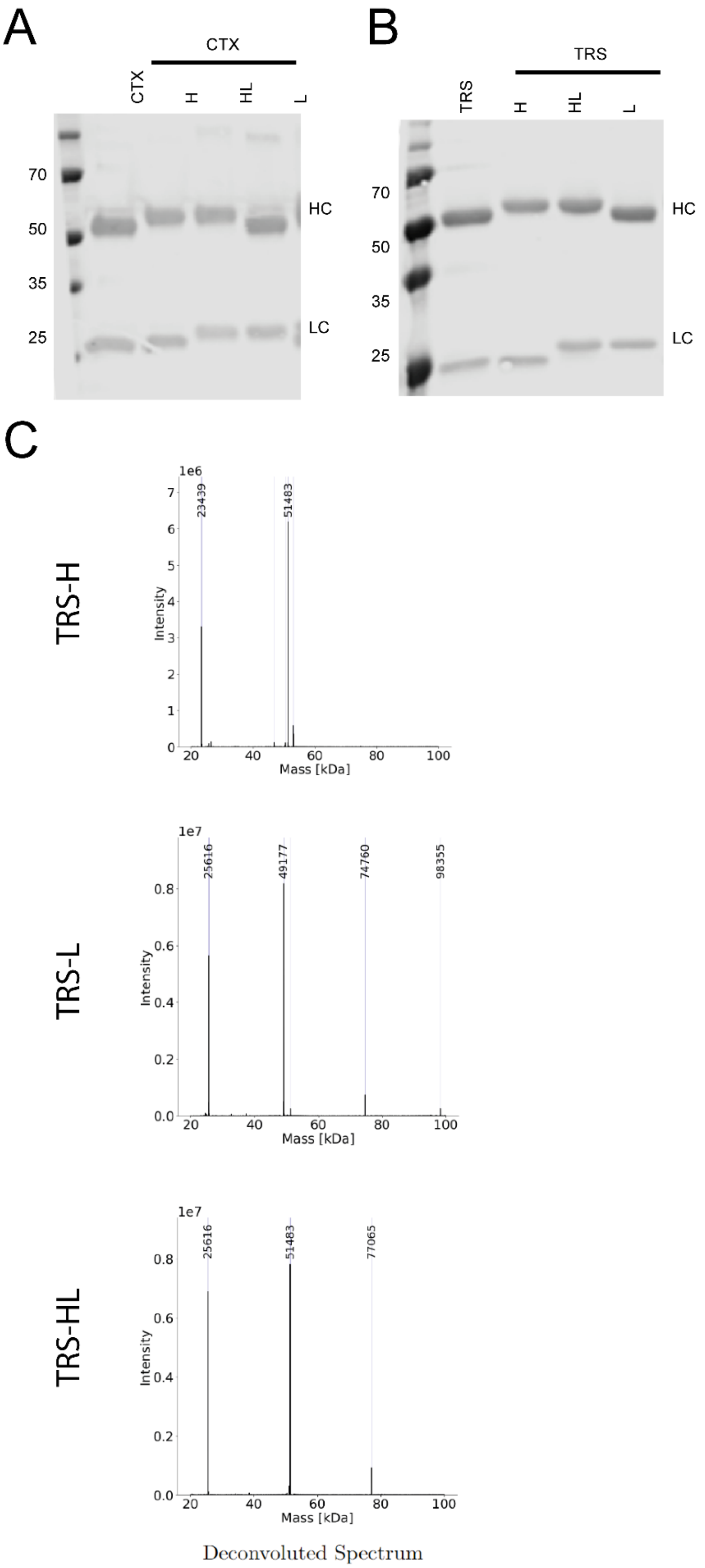

Figure S1: Conjugation analysis of the genetically fused AECs. *SDS-PAGE analysis of the genetic fused AECS compared to the non-modified antibody for* ***(A)*** *CTX and* ***(B)*** *TRS.* ***(C)*** *The presence of the intact epitope was confirmed with MS-spec data.*

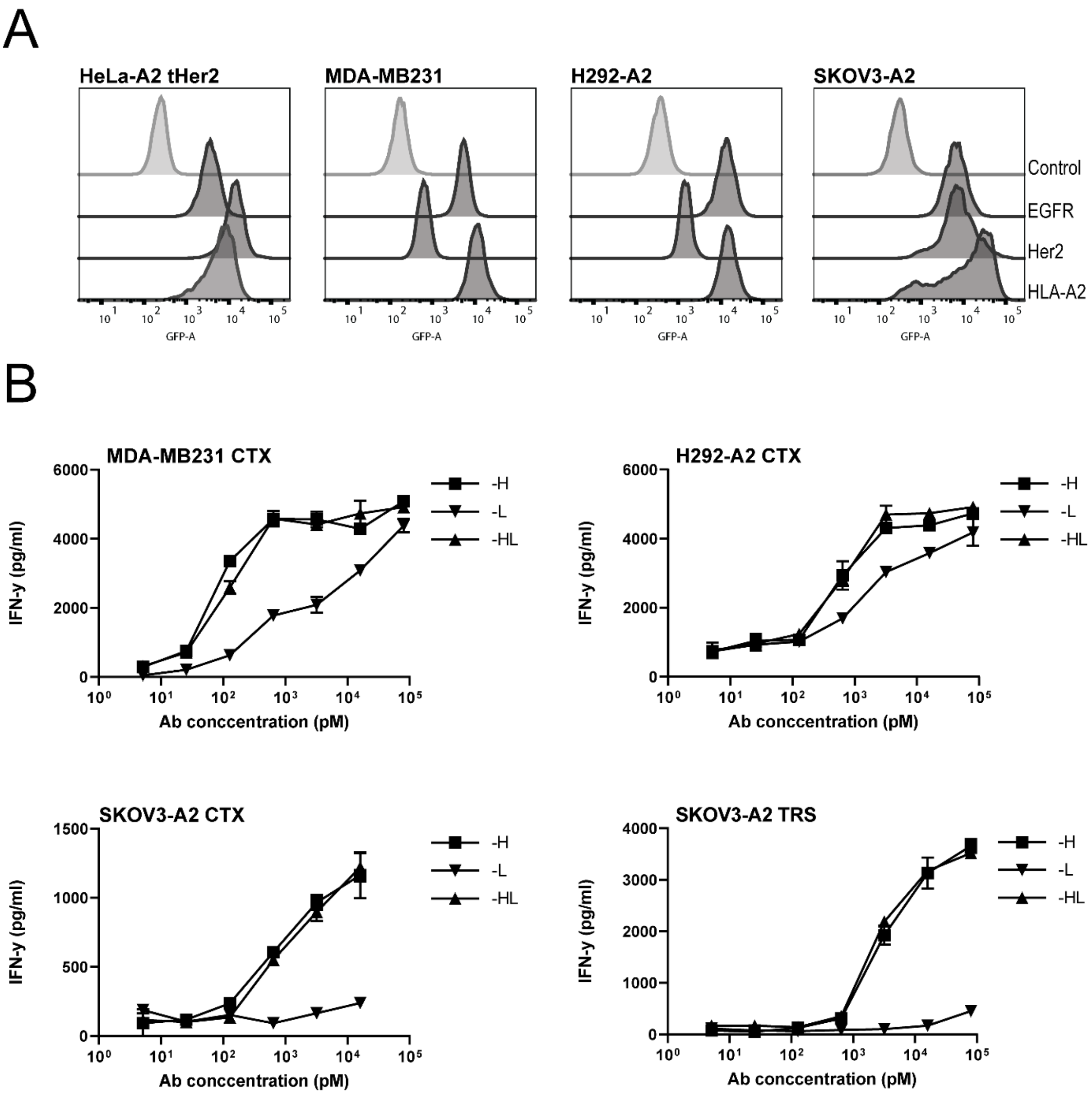

Figure S2: Expression analysis and cocultures on four other cell lines (A) *Expression analysis of EGFR, Her2 and HLA-A2 for the cell lines SKOV3-A2, MDA-MB231 and H292-A2.* ***(B)*** *The results of coculture experiments for CTX-AECs with the SKOV3-A2, MDA-MB231 and H292-A2, and for TRS-AECs with SKOV3-A2. Plotted values are the means of duplicates (SEM) and each graph shows a representative figures of an n=3.*

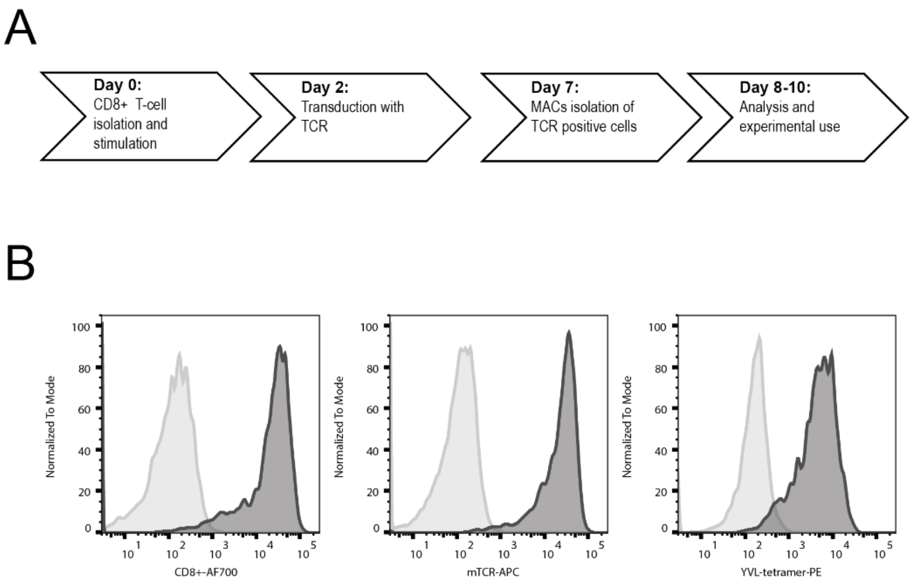

Figure S3: Primary T-cell isolation and transduction with TCR. A) *Schematic overview of how the T-cells are generated* ***B)*** *and an example of the analysis performed on day 10. On day 10 the T-cells were checked for CD8 expression, the expression of the transduced TCR (mTCR), and tetramer binding. The transduced TCR can be distinguished from the endogenous TCR because part of the constant domain contains a mouse sequence.*

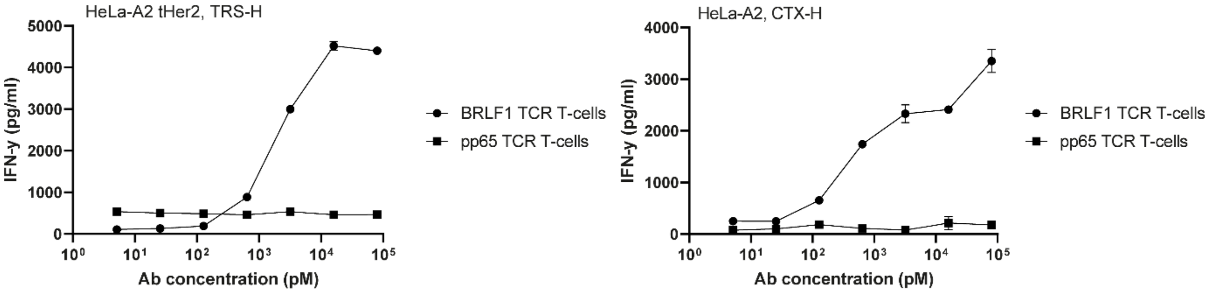

Figure S4: The target cells incubated with AEC-H are not recognized by CD8+ T-cells transduced with non-specific TCR. *HeLa-A2 tHer2 or HeLa-A2 cells were exposed to titrated concentrations of TRS-H or CTX-H, respectively. Followed by a coculture with CD8+ T-cells transduced with either the BRLF1 TCR or a non-specific TCR recognizing the CMV pp65-NLV epitope presented in HLA-A2. T-cell activation was measured with an IFN-γ ELISA.*

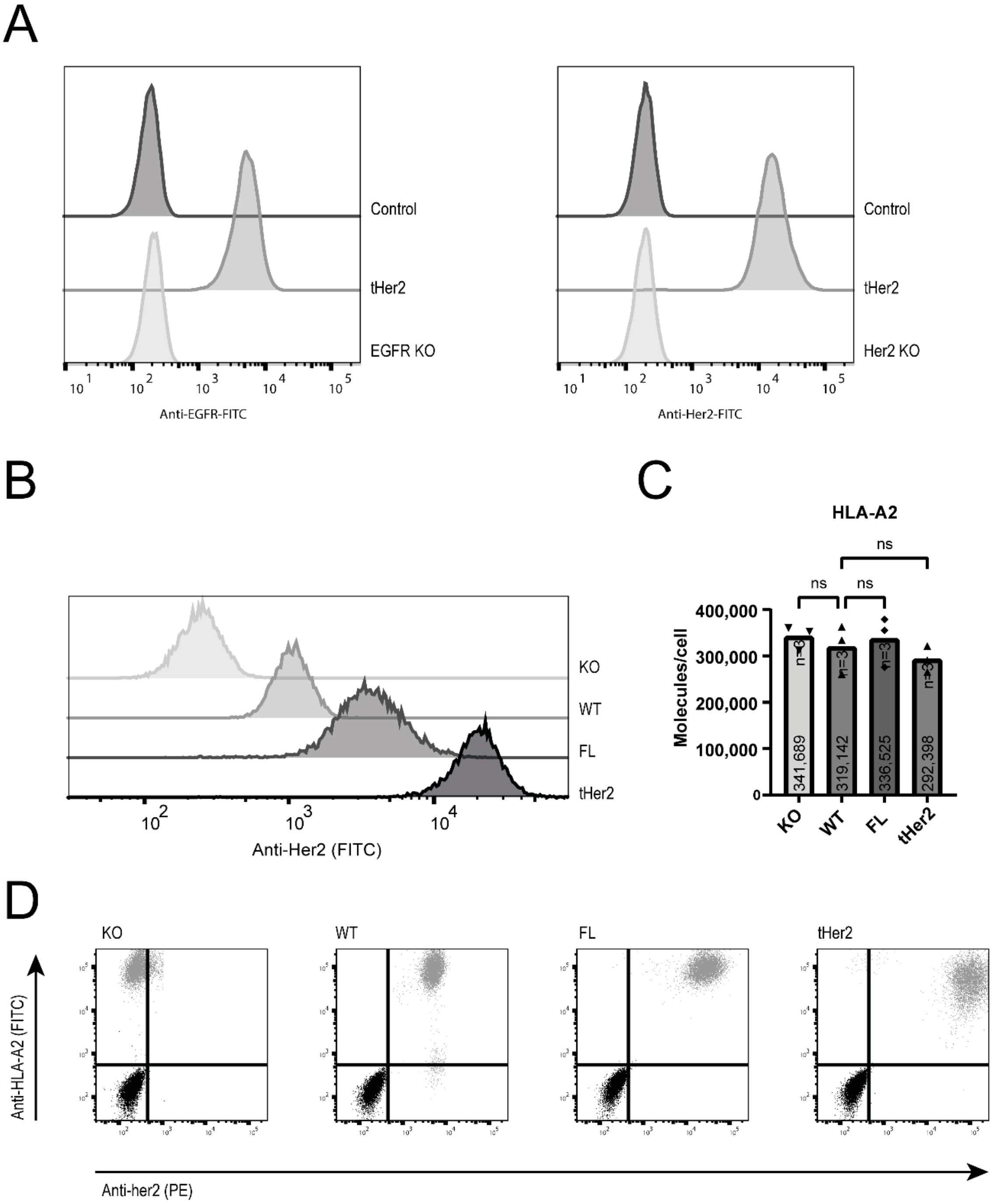

Figure S5: Expression analysis of Her2 and HLA-A2 on four generated HeLa cell lines (A) *FACs analysis of the EGFR and Her2 KO cell lines* ***(B)*** *FACS plots of the different HeLa-A2 cell lines.* ***(C)*** *As a control, the HLA-A2 levels were quantified as all cell lines were originally derived from the HeLa-A2 cell line.* ***(D)*** *FACs analysis of two-colour stained cells using anti-her2 (trastuzumab) and anti-HLA-A2 (BB7.2). As a control (in black) a double anti-human-IgG-PE and anti-mouse-FITC staining was taken along.* ***(C and D)*** *Cells used are Hela-A2 cells with Her2 knockout (KO), HeLa-A2 with wildtype Her2 (WT) expression levels, HeLa-2A KO with full-length Her2 (FL) or lacking the intracellular domain of Her2 (tHer2)*

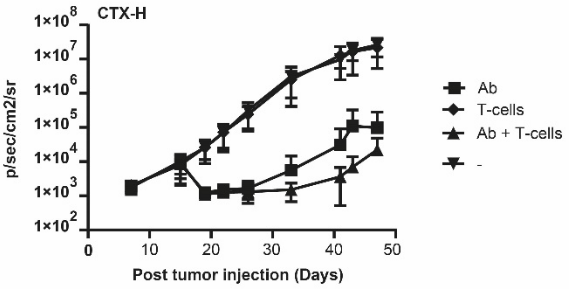

Figure S6: In vivo experiment with antibodies containing the intact Fc-region. *NSG mice were engrafted with U266-tEGFR cells. On day 14, CD8+ T-cells transduced with the BRLF1 TCR were intravenously (i.v) injected, followed by an i.v. injection of the 100 ug CTX-H on day 15 and 18. Tumour growth of the U266-tHer2 was visualized by bioluminescence imaging 1-2 times per week of the ventral side.*

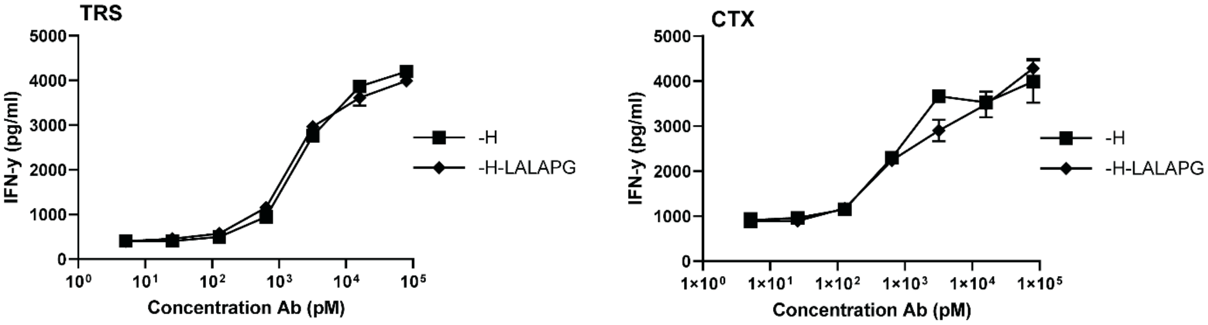

Figure S7: Introducing the LALAPG mutations does not influence T-cell activation. *The cocultures were repeated for both TRS- and CTX-GENH and –GENH-LALAPG. Plotted values are the means of duplicates (SEM) and each graph shows a representative figures of an n=3.*

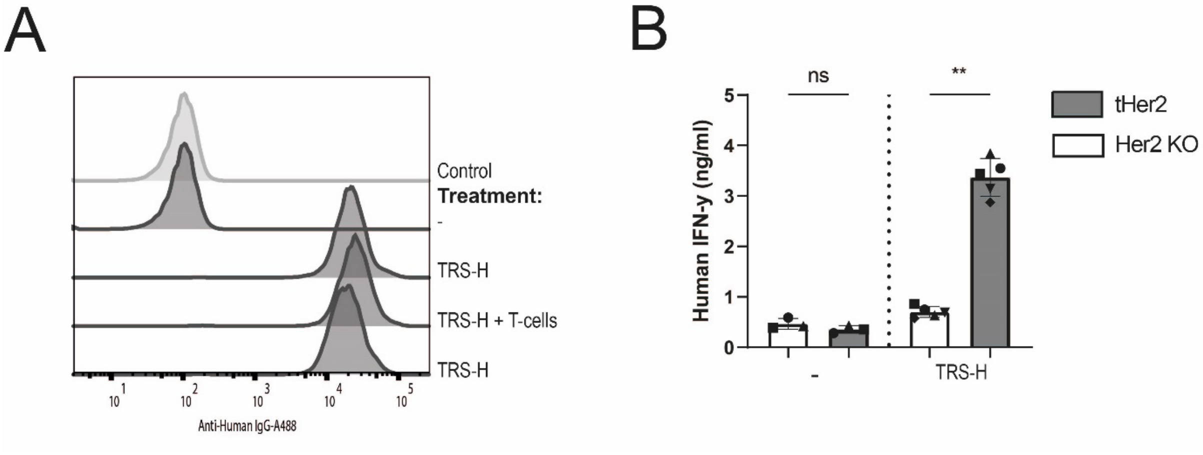

Figure S8: Antibody is still functional and present after the 2nd Ab injection A) *Serum was diluted 1:50 and HeLa-A2 tHer2 cells were incubated with the serum, followed by an incubation with anti-Human IgG-A488 and measured on the FACS.* ***B)*** *The serum was also taken along in a functional assay were HeLa-A2 tHer2 or HeLa-A2 Her2 KO cells were incubated with 2% serum in IMDM. This was washed away after 1,5 hr and cocultured with YVL-specific T-cells. Plotted values are the means of duplicates (SEM) and each dot represents the data of one mouse.*

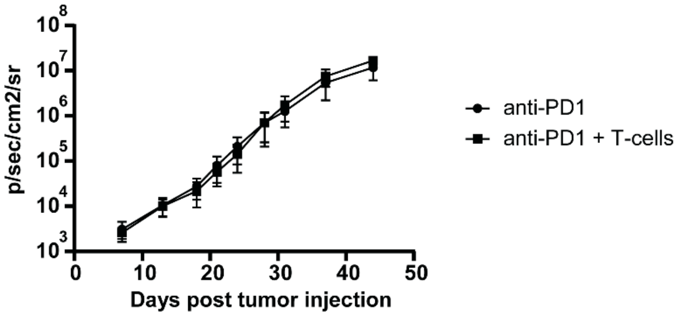

Figure S9: *To rule out a side effect of the anti-PD1 treatment on the virus-specific T-cells and on the tumor growth, 2 groups of mice engrafted with U266-tEGFR (N=4) in a separate experiment received pembrolizumab (anti-PD1) with or without the EBV-TCR T-cells on day 14. No difference in tumor outgrowth was observed.*
